# Determinants of hookah smoking among men in the coffee houses: an application of socio-ecological approach

**DOI:** 10.1186/s13011-020-00305-2

**Published:** 2020-08-24

**Authors:** Fatemeh Bakhtari, Asghar Mohammadpoorasl, Haidar Nadrian, Nader Alizadeh, Leila Jahangiry, Koen Ponnet

**Affiliations:** 1 Health Education and Health Promotion Department, School of Public Health, Tabriz University of Medical Sciences, Tabriz, Iran; 2 Department of Health Education & Promotion, Road Traffic Injury Research Center, Tabriz University of Medical Sciences, Tabriz, Iran; 3 Epidemiology and Biostatistics Department, School of Public Health, Tabriz University of Medical Sciences, Tabriz, Iran; 4 Tabriz Health Services Management Research Center, Tabriz University of Medical Sciences, Tabriz, Iran; 5 Faculty of Social Sciences, imec-mict Ghent University, Ghent, Belgium

**Keywords:** Hookah, Coffee houses, Socio-ecological approach, Men

## Abstract

**Background:**

Tobacco smoking is the second leading cause of death and is closely linked to fatal diseases. Hookah Smoking (HS) is a traditional way to smoke tobacco, especially in the Eastern Mediterranean region that is constantly rising around the world. This study aimed to evaluate the different levels of personal, interpersonal and social HS in Iranian urban men and determine the most important predictors of the levels through applying the socio-ecological approach (SEA).

**Methods:**

This study was conducted in the coffee houses of Hashtrud and Qarah Aghaj counties in East Azerbaijan, Iran. Data collection was conducted from the entire coffee house (*n* = 18) from April to June 2017. Systematic sampling was employed to recruit 266 men in the coffee house. A valid and reliable instrument was used to investigate the frequency of HS and its determinants based on SEA. The SEA consists of three levels: personal (age, education, employment, income, and perceived severity and sensitivity), interpersonal (perceived reward), and social level (social support) intended to assess HS determinants. Hierarchical regression was used to determine the predictive value of SEA levels and frequency of HS.

**Results:**

The mean age of daily hookah smokers (once per day and more than once per day) were (26.8) significantly lower than those (30.4) smokes weekly (once a week or more than once a week). The hierarchical logistic regression model showed that in the first step individual variables significantly predict 25.1% HS. In the second and third level interpersonal and social levels of SEA explained HS 30.1 and 30.8%, respectively.

**Conclusion:**

This study found that age, income, education, and perceived reward were all important factors influencing HS among men youth. Application of SEA to determine the factors associated with HS could contribute in the development of a holistic prevention program.

## Introduction

Hookah Smoking (HS) is a traditional way to smoke tobacco, especially in the Eastern Mediterranean region that is constantly rising around the world [1]. In many Eastern Mediterranean countries, HS in adults has reached 20–30%, and is also rising in younger consumers [2, 3]. According to the WHO, half of hookah smokers die from hookah-related diseases, and about 70% of these deaths occur in developing countries [4]. Evidence suggested that hookah has adverse effects on communicable and non-communicable diseases, thereby intensifying public health issues related to HS [5].

Tobacco smoking is the second leading cause of death [6] and is closely linked to fatal diseases [7]. Given the harmful effects of HS and its increasing association with morbidity and mortality, supporting significance efforts to prevent and reduce of HS should be taken into account [8]. Various studies have highlighted several reasons for HS. Some studies have reported having a hookah smoker in family member and friend are the major causes of HS [1, 9–11]. The others have reported that limited knowledge about the adverse effects of HS [12], social support [9, 13] and perceived sensitivity, severity and reward are the reasons for the tendency to HS [14]. Although each of these studies has addressed a particular aspect of HS, such studies often fail to apply theoretical approach to provide the most important cause of HS in analytical context. To prevent HS, multiple factors need to be in the account when choosing the best strategies to promote HS cessation among adults [15–17]. It is necessary to employ a comprehensive framework to counter all essential elements of the health issue.

In the field of public health, Social Ecological Approach (SEA) describes people’s interactions with their physical and socio-cultural surroundings [18, 19]. SEA incorporates a wide range of influences at multiple levels of variables rather than positing behavior under the influence of personal factors [20, 21]. To promote the health of vulnerable population, it’s need to investigate the influence of multi-predictor approach which incorporates the factors from personal and interpersonal levels to organizational, social and political levels [22].

This study aimed to evaluate the different levels of personal, interpersonal and social HS in Iranian urban men and determine the most important predictors of the levels through applying the SEA.

## Methods

### Study design and participants

This study was conducted in the coffee houses of Hashtrud and Qarah Aghaj counties in East Azerbaijan, Iran. Hashtroud and Qarah Aghaj are the small towns with fewer entertainment opportunities in comparison with the large cities. Coffee houses are available and accessible options to be used as convenient and comfortable places for friendly get-togethers among men in the study setting. In the coffee houses, drinking tea and smoking hookah are common, and thus, men are exposed to HS. Data collection was conducted from the entire coffee house in Hashtroud and Qarah Aghaj from April to June 2017. Systematic sampling was employed to recruit 266 men in the coffee house. The inclusion criteria consisted of being male, age 15 to 35 years, and smoke hookah. Exclusion criteria were: using drugs, narcotics, and substance, being illiterate, and suffering from psychological disorders or mental problems such as depression. Informed written consent was obtained from all participants. The study received ethical approval from the Ethics Committee of Tabriz University of Medical Sciences (NO: IR.TBZMED.REC.1396.175).

### Instrument

We intended to use our questionnaire to gather information from our target population about the potential determinants of HS. The questionnaire included two sections; the first part of questionnaire included an item: “how often do you smoke hookah?” with five possible responses (*once a week or less, more than once a week, once per day, and more than once per day*).

The second section applied SEA that consisted of three levels including personal, interpersonal and social level. The following questionnaires are used to measure the factors in each level:

To assess the factors in the personal level, perceived susceptibility including 5 items and perceived severity including 8 items, each of which had 5 possible answers *(ranging from 1 = strongly disagree to 5 = strongly agree).* The sample item for perceived susceptibility and severity were “Using hookah will increase my chance for getting lung cancer”, and “Smoking hookah will reduce my chance for getting a suitable job”, respectively. The maximum total score for perceived susceptibility and severity were 25 and 40, respectively. The higher the score in scales indicated the higher levels of perceived susceptibility and severity among hookah smokers. The validity and reliability of the scales were well documented in the study from Iran [14]. Also, demographic characteristics including age, educational level, marital status, employment, and income status were measured in personal level.

To assess the factors in the interpersonal level, perceived internal and external rewards were measured applying a nine-item scale. The sample item for perceived internal rewards was “By hookah smoking, I feel like a grown-up and feel like a man”, each of which had five possible answers (ranging from 1 = strongly disagree to 5 = strongly agree). The higher the score, the higher levels of internal and external rewards were perceived by the individuals to smoke hookah. Validity and reliability of the scale has been confirmed in a previous study [14].

Social factor was investigated by measuring perceived social support (14 items). The sample items for perceived social support were items contain friends’ accompaniment during HS, friends’ encouragement to go to the coffee houses, and their invitation to go to a coffee house to smoke hookah. The response format for the items was based on a 5-point Likert-type scale (from 1 = never] to 5 = always). Content validity index (CVI) and content validity ratio (CVR) values for the scale were 90.64 and 74.42, respectively, and the alpha Cronbach’s coefficient was 0.72.

### Data analysis

SPSS version 23 was used to analyze the data. Descriptive statistics were calculated to determine mean and standard deviation in quantitative data and frequency and percentage in qualitative data. The association between frequency of HS with demographic variables and SEA levels were analyzed using one-way ANOVA and linear regression. Chi-square tests were used for categorical variables, frequencies and percentages. Moreover, Hierarchical regression was used to determine the predictive value of SEA levels and frequency of HS. In all analyses *P* < 0.05 was considered as significant level.

## Results

In total, 266 HS people participated in this study. The demographic characteristics of the participants as well as their associations with frequency of HS are displayed in Table 1. The mean age of daily hookah smokers (once per day and more than once per day) were significantly lower than those smokes weekly (once a week or more than once a week). Hookah smoking is significantly more common (18% once per day) among single people. Most of our participants had higher education and the rate of HS was significantly higher among people with diploma than the others.

**Table 1:** Demographic characteristics and their associations with frequency of hookah consumption among the participants (*n* = 266)

	Mean (SD)	Frequency of hookah consumption				
characteristics		Once a week or less	More than once a week	Once per day	More than once per day	*P*-value
**Age (years)**	37.9 ± 10.96	30.4 ± 6.7	30.9 ± 5.1	26.43 ± 5.7	26.8 ± 4.8	< 0.001
**Marital status**						
Married	139 (52.3)	31 (22.2)	60 (43.2)	36 (25.9)	12 (8.6)	0.001
Single	127 (47.7)	23 (18.1)	31 (24.4)	50 (39.4)	23 (18.1)	
**Education level**						
Secondary school	18 (6.8)	0 (0)	7 (38.9)	8 (44.4)	3 (16.7)	0.031
Diploma	127 (47.7)	22 (17.3)	38 (29.9)	50 (39.4)	17 (13.4)	
University	121 (45.5)	32 (26.4)	46 (38.0)	28 (23.1)	15 (12.4)	
**Employment status**						
Employed	225 (84.6)	45 (20.0)	84 (37.3)	67 (29.8)	29 (12.9)	0.06
Unemployed	41 (15.4)	9 (22.0)	7 (17.1)	19 (46.3)	6 (14.6)	
**Income** (10 million Rials per month)						
Less than 1	54 (20.3)	20 (9.3)	22 (10.2)	6 (2.8)	5 (2.3.)	0.67
1–2	131 (49.2)	39 (18.1)	55 (25.6)	14 (6.5)	16 (7.4)	
More than 2	42 (15.7)	16 (7.5)	17 (7.5)	5 (2.3)	0	

The descriptive statistics of hookah smoking factors in individual (Perceived sensitivity and Perceived severity), interpersonal level (perceived reward), and social level (social support) are shown in Table 2. Perceived severity was lower among people smoking hookah less frequently (once a week or less), and it was significantly higher among those who smoked hookah more frequently (more than once per day).

**Table 2:** Descriptive statistics for Socio-ecological Model levels and hookah consumption

	Mean (SD)	Once a week or less	More than once a week	Once per day	More than once per day	*P*-value
**personal level**						
Perceived sensitivity	19.98 (3.90)	12.1 ± 1.2	12.1 ± 1.2	12.4 ± 2.7	12.3 ± 2.6	0.73
Perceived Severity	9.56 (2.78)	25.7 ± 2.7	24.3 ± 3.0	26.3 ± 3.7	25.8 ± 3.2	0.001
**Inter-personal Level**						
Perceived reward	23.60 (5.72)	32.3 ± 5.9	37.1 ± 4.4	31.7 ± 5.3	32.4 ± 5.1	< 0.001
**Social level**						
Social support	36.59 (6.28)	36.8 ± 4.0	34.9 ± 4.5	36.9 ± 6.0	37.4 ± 6.5	0.02

*SEA Socio-ecological Approach

In the hierarchical logistic regression model (Table 3), the first step assess the influence of individual level (perceived severity, perceived sensitivity, age, education, being employed or unemployed, and income) on HS. The results showed that individual variables predict up to 25.1% of the HS. Among these variables, income, age, and education were significant predictors for HS. The second step including interpersonal level (reward) added significantly to the model. In this step, the predictive effect of the individual and interpersonal levels was investigated and the results showed that the factors predict up to 30.1% of HS. In this step, perceived rewards, income, age, and education significantly contributed to HS. In the third step, the predictive effect of individual, interpersonal and social factors were examined and the results showed that these factors predicted about 30.8% of HS.

**Table 3:** Hierarchical Regression analysis to predict Hookah Smoking related factors according to SEA

level	Step/variable	*B (Step1)*	SE	*P*-value	*B (Step2)*	SE	P- value	*B (Step3)*	SE	*P*-value
Personal	Perceived severity	.115	.009	.059	.020	.009	.761	.027	.010	.701
	Perceived sensitivity	.085	.013	.159	.035	.013	.558	.036	.013	.547
	Age	−.217	.006	.002	−.213	.006	.002	−.216	.006	.002
	Education	−.136	.049	.028	−.166	.048	.007	−.164	.048	.007
	Employed or unemployed	−.075	.260	.212	−.090	.254	.126	−.108	.259	.073
	Income	−.265	.048	.000	−.210	.048	.002	−.197	.049	.004
Inter-personal	Perceived reward	–	–	–	−.232	.006	.001	−.251	.006	.001
Social	Social support	–	–	–	–	–	–	.009	.007	.891
	*R*^*2*^	*.251*			*.301*			*.308*		
	*Cumulative R*^*2*^	*.251*			*.552*			*.860*		
	*P value*	*0.0001*			*0.0001*			*0.0001*		

## Discussion

This study evaluated a SEA for prevention and control of HS among men youth. In this study SEA includes personal (perceived severity, perceived sensitivity, age, education, income, and employment status), interpersonal (perceived reward), and social factors (social support).

This study found that age, income, education, and perceived reward were all important factors influencing HS among men youth. Application of SEA to determine the factors associated with HS could contribute in the development of a holistic prevention program. Similar studies have shown that the perceived reward of smoking, such as the feeling of greatness and pride are the most important predictors of HS [14]. As young people are particularly susceptible to social influence and are more likely to engage in high risk behaviors in the presence of their friends [23]. For young people, groups of friends and their activities are very important and they consistently getting along with them and exhibit the same behavior. By the other words, social and physical environments are one of the main factors in encouraging to HS [24]. This happens in the coffee house, the most common place offering HS. Therefore, creation of healthy and friendly tobacco-free environments may help in reducing HS.

The rate of HS per day in coffee houses has been reported 82% [9]. Rezk-Hanna et al. (2014) in southern California reported that 36.3% of their research participants smoked hookah four times or over per week [25], which was less than of our findings. This may be due to the sociocultural differences between the settings of the studies. The review studies conducted in the Eastern Mediterranean region noted that a decline in the age of first HS among adolescents and university students was observed [26, 27]. A study suggested that if one does not consume tobacco up to 26 years old, he/she most probably won’t use it in the future [24]. Therefore, it seems necessary to plan preventive programs against hookah smoking in early stage of adolescence. In the present study, the income was the predictor of HS. Studies have shown that at a younger age, when a person is employed and has purchasing power, his/her rate of HS is high, but when the person is unemployed and has no income, he/she is likely to expend less money for HS [28]. In addition, educational level was also drawn as a predictor for HS in our study. People with the higher education smoke hookah less frequently [29]. However, because people of younger ages usually have lower education level and lower income amount [30, 31]., it may be necessary to compare our results with those of additional studies on different age, income amount, and occupation groups of people.

Similar to other study, the present study showed that perceived severity was significantly associated with HS [14, 32]. Therefore, it is necessary to consider the interventions for increasing perceived severity, especially in frequently hookah-smokers. Social support is one of the predictor that can contribute positively to improve performance of a behavior. As hookah smoking is a social behavior social support may increase the probability of HS behavior [16, 33]. Considering the role of personal, interpersonal, social and environmental levels in HS and their synergistic effect to increase its use, interventional programs need to start from earlier age, and incorporate all the levels of SEA.

In addition, given the youth spend their leisure time in coffees, policymakers need to develop interventions to create alternative recreational sites, and also the owners of the coffee houses should provide healthy alternative materials such as healthier foods or healthier recreations that appropriate to regional Iranian culture.

As a limitation, this study was conducted in coffee house that hookah is available, so it should be investigated more cautiously. This study was the first study in Iran that simultaneously investigate personal, interpersonal and social environments related to HS and also determined related predictors through hierarchical regression analysis.

## Conclusion

It was concluded that SEA was a useful approach in predicting HS. Frequency of HS was high special in lower age. Therefore, conducting educational intervention efforts at personal, interpersonal level, and physical environmental change of coffee houses are recommended.

## Data Availability

The data collection tools and datasets generated and/or analyzed during the current study are available from the corresponding author upon reasonable request.

## Abbreviations

HS: Hookah Smoking
SEA: Socio-ecological approach
CVI: Content validity index
CVR: Content validity ratio
